# Isolation and Genetic Characterization of Puumala Orthohantavirus Strains from France

**DOI:** 10.3390/pathogens10030349

**Published:** 2021-03-16

**Authors:** Johann Vulin, Séverine Murri, Sarah Madrières, Maxime Galan, Caroline Tatard, Sylvain Piry, Gabriele Vaccari, Claudia D’Agostino, Nathalie Charbonnel, Guillaume Castel, Philippe Marianneau

**Affiliations:** 1 Laboratoire de Lyon, ANSES, Unité de Virologie, 69007 Lyon, France; severine.murri@anses.fr (S.M.); sarah.madrieres@anses.fr (S.M.); philippe.marianneau@anses.fr (P.M.); 2 CBGP, INRAE, CIRAD, IRD, Institut Agro, Univ Montpellier, 34000 Montpellier, France; maxime.galan@inrae.fr (M.G.); caroline.tatard@inrae.fr (C.T.); sylvain.piry@inrae.fr (S.P.); nathalie.charbonnel@inrae.fr (N.C.); guillaume.castel@inrae.fr (G.C.); 3 Department of Food Safety, Nutrition and Veterinary Public Health, Istituto Superiore di Sanità, 00161 Rome, Italy; gabriele.vaccari@iss.it (G.V.); claudia.dagostino@iss.it (C.D.)

**Keywords:** Hantavirus, Puumala, isolation

## Abstract

Puumala orthohantavirus (PUUV) causes a mild form of haemorrhagic fever with renal syndrome (HFRS) called nephropathia epidemica (NE), regularly diagnosed in Europe. France represents the western frontier of the expansion of NE in Europe with two distinct areas: an endemic area (north-eastern France) where PUUV circulates in rodent populations, with the detection of many human NE cases, and a non-endemic area (south-western France) where the virus is not detected, with only a few human cases being reported. In this study, we describe the different stages of the isolation of two PUUV strains from two distinct French geographical areas: Ardennes (endemic area) and Loiret (non-endemic area). To isolate PUUV efficiently, we selected wild bank voles (*Myodes glareolus*, the specific reservoir of PUUV) captured in these areas and that were seronegative for anti-PUUV IgG (ELISA) but showed a non-negligible viral RNA load in their lung tissue (qRT-PCR). With this study design, we were able to cultivate and maintain these two strains in Vero E6 cells and also propagate both strains in immunologically neutral bank voles efficiently and rapidly. High-throughput and Sanger sequencing results provided a better assessment of the impact of isolation methods on viral diversity.

Orthohantaviruses represent an increasing threat to humans due to their worldwide distribution, the increase in the number of infections and the emergence or re-emergence of new viruses [1]. Puumala virus (PUUV) is the main orthohantavirus circulating in France. This tri-segmented, enveloped RNA virus, hosted by a rodent, the bank vole (*Myodes glaerolus*), causes a mild form of haemorrhagic fever with renal syndrome (HFRS) called nephropathia epidemica (NE) [2]. About 100 cases of NE are reported annually in north-eastern France [3].

In France, PUUV sequences have been studied in bank vole samples [4] and more recently in human samples [5], but these studied strains have never been cultured or maintained until now. Considering the adaptation capacity of PUUV to cell culture conditions and changes in infectivity induced by cell culture passages [6], obtaining a “wild-type” virus is a crucial step towards a better understanding of the biology of this virus. Unfortunately, due to its slow growth in cell culture, PUUV is often very difficult to isolate [7].

In this study, we applied several methods that enabled the identification of animals carrying the virus, to isolate and cultivate the corresponding strains and to study the effect of the isolation protocol on virus diversity. All of these methods are shown in detail in Supplementary Information S1.

Firstly, we determined a method for selecting bank voles containing live virus from which to isolate PUUV strains. Seto et al. [8] isolated PUUV from bank voles that harboured the orthohantavirus nucleocapsid protein (NP) in their lungs, but showed no antibodies against PUUV. This detection of infection coupled with the absence of antibodies probably corresponds to an active viraemic phase. We adapted the Seto et al. approach by screening our collections of bank vole samples (composed of voles captured in various French forests over the last 10 years [4,9]) for seronegative animals with high amounts of viral RNA in their lung tissue. Serum samples were assayed using IgG ELISA and viral RNA was extracted from lung homogenates. More specifically, we screened bank voles captured in two distinct French geographical areas: (i) an NE endemic area: Ardennes, where PUUV circulates in rodent populations and where many cases of human NE are detected; (ii) a non-endemic zone: Loiret, where PUUV circulates in rodent populations and where no or very few cases of NE are diagnosed [4]. In our previous studies, we showed that the virus from these two areas represents two distinct phylogenetics clusters [4,10,11]. We selected seven bank voles captured in Ardennes during autumn 2011 (out of a total of 201 animals captured during this trapping session) and one bank vole captured in Loiret in summer 2014 (out of 44 animals). For our first isolation assays, we used a sample from Ardennes named Hargnies/2011 with the smallest cycle threshold value (Ct; i.e., 18.90 cycles) and a lung tissue sample from Loiret named Vouzon/2014 that showed a Ct of 23.65 cycles.

From these two animals, we tried to isolate live viruses from lung homogenates (5% *w*/*v*). The conditions that led to the in vitro isolation of the two viral strains consisted of the use of the lung homogenate centrifuged (300 rpm for 10 min), not filtered, diluted to 10^−1^ and applied to low-passaged Vero E6 cells (16 passages) in DMEM (Dulbecco′s modified Eagle′s medium) supplemented with 5% foetal calf serum (Gibco) and cultured to hyper-confluence (1 × 10^6^ cells for one well in P6). It was necessary to carry out three successive passages to reach a sufficient quantity of live viruses. These passages were carried out using “co-culture”: a quarter of the initially infected cells were transferred to a culture of non-infected cells (2 × 10^5^ cells/well in P6) accompanied by 500 µL of the supernatant from the infected culture added to 2 mL of fresh media. 

The viral load measured in the supernatant of Vero E6 cells increased substantially: −15.2 Ct and −12.8 Ct for Ardennes-Hargnies and Loiret-Vouzon, respectively (Figure 1A). Moreover, at the end of the cell isolation, viral titre was 1.6 × 10^4^ focus forming units (FFU)/mL and 1.5 × 10^4^ FFU/mL for Ardennes-Hargnies and Loiret-Vouzon, respectively. Even if the final viral titers were similar, the viral titer and viral RNA quantities were slightly different during the two first passages. After titration, their focus sizes were different to that of the PUUV control (Sotkamo strain) serially passaged in Vero E6 cells (Figure 1B). The focus size of cultivated viruses was smaller (in particular for Loiret-Vouzon) than Sotkamo foci. Phenotypic variations have already been described for PUUV titration assays [12]. Altogether, these results show that we were able to select viral isolates from natural host tissue and cultivate them in cell culture. 

Furthermore, we carried out an in vivo isolation process that consists of biological assays on bank voles that were maintained at the ISS Institute (Rome, Italy). We used the same lung homogenates already used for the in vitro isolation process. We performed a subcutaneous injection of lung homogenate (5% *w*/*v*) on two animals for each strain. None of the inoculated animals showed clinical evidence of viral infection. Seven days after infection, we collected blood samples via retro-orbital sinus puncture and carried out PUUV serological detection and viral RNA analyses. The animals’ sera (pre-diluted at 1/10) were screened using IgG ELISA with PUUV recombinant nucleocapsid (N-PUUV) protein and negative controls. Samples were considered positive if the optical density (OD) was greater than 0.100. We did not detect any anti-PUUV antibodies (Table 1), but we found viral RNA in all sera analyzed. Viral loads were similar regardless of the PUUV strain (Ardennes-Hargnies and Loiret-Vouzon), indicating that our protocol led to the effective transmission of PUUV in these animals. Two days later, at nine days post-infection (D9), we euthanized the four bank voles by cervical dislocation. We found that all animals had seroconverted, with a higher antibody titre observed for the Loiret-Vouzon strain (1/800) than for the Ardennes-Hargnies strain (animal 1: 1/200 and animal 2: 1/100). The RNA viral loads in the sera had already decreased in all four animals analysed, but were still positive. We also analysed the RNA viral loads in the lungs and liver, i.e., organs in which PUUV antigens have previously been detected [13,14]. We found high viral RNA loads (Table 2), which confirmed the effective infection of all animals by PUUV.

Molecular analyses were carried out as already described [7] to assess the PUUV levels during bank vole infection. RNA was extracted from lung tissue samples of positive bank voles (natural populations and experimentally infected) and from the supernatant of Vero E6 cells, then sequenced to obtain the complete coding sequence of the S and M segments (for a list of sequences and accession numbers, see Supplementary Table S2). In order to provide a better assessment of the impact of isolation methods on intra-host viral diversity, amino-acid sequences were compared between naturally infected bank vole (in natura), cell-cultivated virus (in vitro) and experimentally infected bank vole (in vivo) for each strain (Ardennes-Hargnies or Loiret-Vouzon). For the M segment (3444 bp for coding region), the PUUV nucleotide sequences from Ardennes-Hargnies or Loiret-Vouzon were 100% identical in the three conditions analyzed by strains. For the S segment (1304 bp for coding region), the amino-acid sequences from Ardennes-Hargnies or Loiret-Vouzon revealed one mismatch between the sequences from bank vole (in natura and in vivo) and in vitro (Figure 2). For Ardennes-Hargnies, bank voles (in natura and in vivo) showed an arginine at codon 63 (R63), whereas the virus cultivated in cells (in vitro) presented a glutamine (Q63). For Loiret-Vouzon, a difference was found in position 28 with an alanine residue (A28) for bank voles (in natura and in vivo) and serine (S28) for in vitro. It is important to note that these two mutations are located in the major antigenic domain (MAD) previously described [15]. Therefore, the in vitro isolation protocol seemed to have influenced the amino-acid sequences of both PUUV strains.

We then performed HTS analyses to investigate these genetic variations further. For the method details, see Supplementary Information S3, S4, S5 and S6 [16,17,18,19,20,21]. More specifically, we assessed whether the different isolation protocols maintained the initial intra-host viral diversity detected in the original samples (from which the strains were isolated) presented as “in natura” results below. We thus characterized the viral variants pool for the three viral “sources”: in natura, in vitro, and in vivo. Two indices were used to compare the viral diversity between the two isolation protocols and the two strains: the number of polymorphic sites [22,23] and the mean percent complexity (the number of unique sequence reads/total reads × 100) calculated on the 10 amplicons [24]. A Kruskal–Wallis test followed by a Dunn multiple comparison test was conducted to compare the mean percent complexity between strains of different origins (in natura, in vitro or in vivo). For both propagation processes—in vivo and in vitro—the RNA viral sequences discussed after came from endpoint processes (biological assay at D9 for in vivo and D14 of 3rd passage for in vitro).

In natura, the intra-host viral diversity of the Ardennes-Hargnies and Loiret-Vouzon strains were similar. We found no difference in the number of polymorphic sites or in the mean percent complexity between areas. After the in vitro isolation process, one variant of the Ardennes-Hargnies strain (R63), mostly found in natura, switched to another major variant, such as Q63. Nevertheless, R63 was still present at a high frequency (about 23%) instead of a viral diversity decreased after the cell culture passages, regardless of the diversity index considered. The Loiret-Vouzon strain, as observed with the Sanger method, showed a change in the major variant from A28 to S28. However, HTS results were contradictory: the number of polymorphic sites increased, but the mean percent complexity remained stable. The in vitro isolation process lead to a significant difference in viral diversity between PUUV strains, but not in the same way for both strains (Figure 3A). 

Regarding the in vivo isolation process, we only analyzed lungs at endpoint of biological assay (D9). We did not perform any kinetic or multi-organ analyses. We detected a lower number of polymorphic sites compared with the natural strains. However, we found no significant difference in the mean percent complexity between PUUV strains or in in natura and in vitro conditions (Figure 3B). The sequence of the majority variant did not differ between these two conditions, regardless of the isolation protocol considered. Results were similar in bank voles infected with the same strain.

## Conclusions

In this study, we were able, for the first time, to isolate and maintain in cell culture two PUUV strains from two distinct French areas. Molecular analyses of the S and M segments of PUUV originating from natural isolates, from experimentally infected bank voles and from cell cultures revealed only one amino-acid mismatch for the Ardennes-Hargnies and Loiret-Vouzon strains. Both mismatches were identified in cell culture. HTS confirmed this result, thereby leading to the definition of a “bank vole” and a “cell culture” molecular profile. Having these two types of PUUV wild strain cultures is an important asset. Cell-adapted strains provide well-characterized viruses that can be used for antiviral candidate studies or cell-level experiments to assess the role of apoptosis, PUUV propagation or control [25]. Furthermore, wild strains maintained in their natural host can help contribute to improving our knowledge of the PUUV ecology and evolution [26]. The bank voles used in the in vivo experiments came from a “neutral” origin, i.e., not from Ardennes or Loiret. For the moment, bank vole-PUUV interactions are poorly documented, but may be a key point in viral diversity.

Previous studies showed that there is significant inter-regional viral genetic diversity [4,5,27], which may explain, at least in part, the regional differences in NE epidemiological status in France. Here, we showed that the viral diversity of PUUV circulating in the NE endemic area (Ardennes) and in the NE non-endemic area (Loiret) evolves differently when passaged on Vero E6 cells. Further research dedicated to the characterization of the now-available French Puumala strains will help further our knowledge on the NE epidemiological situation.

## Figures and Tables

**Figure 1:**
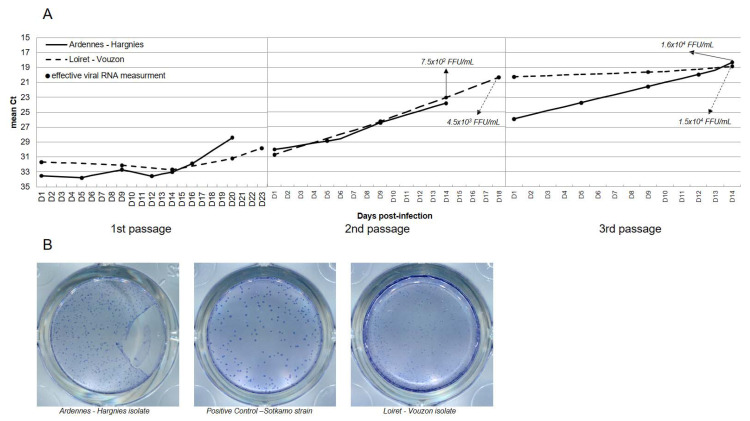
PUUV isolation with Vero E6 cells. Kinetics of PUUV RNA quantity during isolation process (**A**). Circles represent supernatants sampling. Picture of viral titration results (**B**) for Hargnies and Vouzon compared to Sotkamo referential strain.

**Figure 2:**
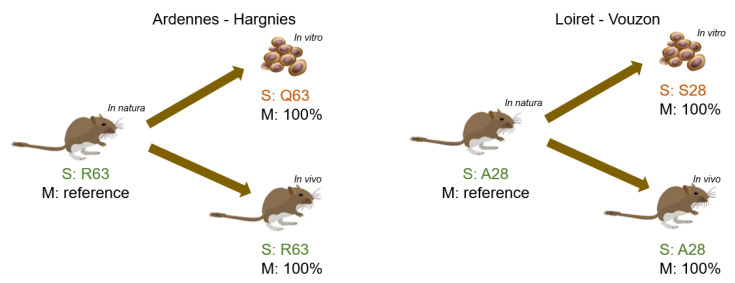
Comparison of S and M segment amino-acid sequences. Synthesis of Sanger sequencing of S and M segments for natural isolates (in natura) (sequence as reference), cell cultivated viruses (in vitro) and experimentally infected bank voles (in vivo). Bank vole and cell drawings were designed by brgfx/Freepik.

**Figure 3:**
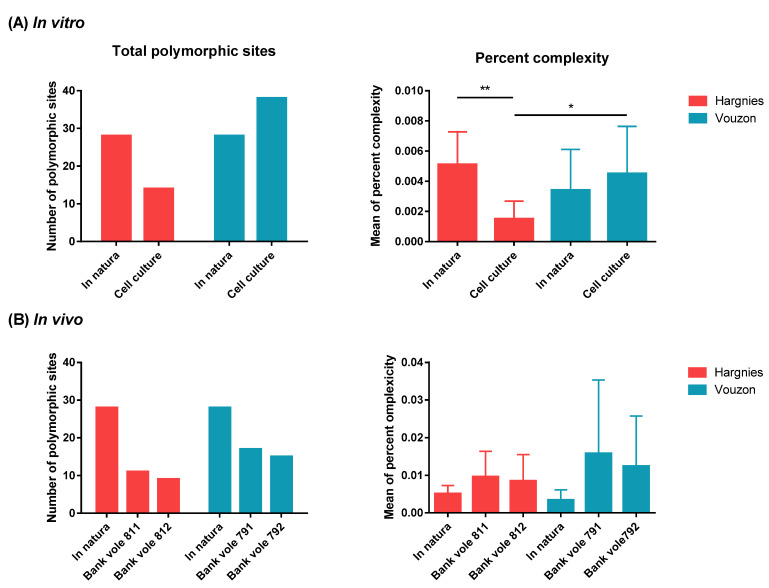
Comparison of viral diversity after (**A**) in vitro and (**B**) in vivo isolation process. *p*-values were determined using the Kruskal-Wallis test (*, *p* < 0.05; **, *p* < 0.01), X^2^ = 13.27, *p* = 0.0041, n = 40.

**Table 1:** Serological results of bank vole experimentally infected with lung homogenates from animals trapped from Hargnies and Vouzon.

Capture Area	Day Post-Infection	Animal Number	Antibody IgG-NPuu		
			IgG-ELISA		Titration
			OD	Conclusion	Last + Dilution (OD)
Ardennes-Hargnies	**D7**	811	0.090	-	ND
	**D7**	812	0.058	-	ND
	**D9**	811	0.140	+	200 (0.102)
	**D9**	812	0.117	+	100 (0.104)
Loiret-Vouzon	**D7**	791	0.032	-	ND
	**D7**	792	0.029	-	ND
	**D9**	791	0.230	+	800 (0.105)
	**D9**	792	0.227	+	800 (0.106)

OD: Optical Density. ND: Not Defined.

**Table 2:** RNA detection results of bank vole experimentally infected with lung homogenates from trapped animals at Hargnies and Vouzon.

Strain	Day Post-Infection	Animal Number	RT-PCR (Mean Ct)			
			Lung	Liver	Sera-Day 7	Sera-Day 9
Ardennes-Hargnies	D9	811	16.5 ± 0.13	19.7 ± 0.12	29.2 ± 0.07	32.1 ± 0.05
		812	19.1 ± 0.00	20.0 ± 0.08	30.6 ± 0.11	32.7 ± 0.11
Loiret-Vouzon	D9	791	19.0 ± 0.05	21.0 ± 0.17	30.7 ± 0.20	31.0 ± 0.04
		792	19.1 ± 0.06	20.8 ± 0.07	29.9 ± 0.19	30.5 ± 0.00

## Data Availability

Not applicable.

## Supplementary Materials

The following are available online at https://www.mdpi.com/2076-0817/10/3/349/s1, Supplementary information S1: methods description, Table S2: PUUV sequences obtained in this study, Table S3: Sequences of primer cocktails used in Miseq, Supplementary information S4: Library preparation adapted from Galan et al. 2018, Supplementary information S5: Definition of the 0.32% threshold, Supplementary information S6: Means number of read used polymorphic sites quantification.
