# Supplementary material for: Isolation and Genetic Characterization of Puumala Orthohantavirus Strains from France
Source: Pathogens. 2021 Mar 16;10(3):349. doi: 10.3390/pathogens10030349 (PMC7999636; doi:10.3390/pathogens10030349)
Supplement: Supplementary file 1 [file pathogens-10-00349-s001.pdf]

Supplementary material

Supplementary information S1: methods description

Screening methods

To screen captured bank voles, serum samples were assayed using IgG ELISA as already described (9) Shortly, animals sera (pre-diluted at 1/10) were screened using IgG ELISA with PUUV recombinant nucleocapsid (N-PUUV) protein and negative controls. Samples were considered positive if the optical density (OD) was greater than 0.100. Viral RNA was extracted from lung homogenates using the QIAamp Viral Mini Kit according to the manufacturer's instructions (Qiagen). Quantitative RT-PCR was performed with 2.5 µL of viral RNA amplified using the SuperScript III One-Step RT-PCR system with Platinum Taq High Fidelity (Invitrogen) on a LightCycler 480 (Roche), see our previous study (28) for more details.

In vitro isolation assay

Once the animals carrying the live virus have been identified we tested two way of isolation: cell culture (*in vitro*) and biological assays on bank voles (*in vivo*).

For *in vitro* way, we tried many cell culture conditions, such as centrifugation (with or without), filtration (0.22 µm) and dilution (10-1 to 10-4) for homogenate preparation; cell confluence and numbers of cell passages before infection (16 or 43) for cell culture and different infection methods between the three passages before isolation (supernatant, transposition or co-culture). The *in vitro* isolation process was monitoring at different times by evaluation of viral titer.

Titration assay

Titration assay was carried out as follow: the supernatant VeroE6 cells cultures were serially diluted in DMEM medium and inoculated onto VeroE6 cells in 24-well plates for a plaque assay. 14 days after infection, cells were fixed with 4% PFA solution for 20-30 minutes. Cells were washed twice with PBS and permeabilized with Triton X100 diluted at 0.5% in PBS for 10 minutes. After 3 washes in PBS, the primary antibody (1/100) raised against Puumala's nucleoprotein (anticorps en ligne.fr – ref ABIN 111531) is incubated during 1 hour at 37 °C. Cells were washed twice with PBS and stained using secondary antibody anti-mouse IgG horseradish peroxidase (HRP)-linked whole antibody (Sigma, #A9044) at 1:1000 in PBS for 1 hour at 37 °C before being visualized by 15 min incubation at room temperature with TrueBlue substrate (Eurobio).

In vivo isolation method

For biological assays on bank voles we used the same lung homogenates already used for the in vitro isolation process. We performed a subcutaneous injection of lung homogenate (5%

w/v). The viral RNA obtained after in vitro and in vivo isolation ways was sequenced by Sanger method and high-throughput sequencing (HTS) as described below.

Sangers method

RNA was amplified using RT-PCR and nested PCR as already described (4). PCR products were sequenced using the Sanger method. The S and M sequences from Ardennes - Hargnies and Loiret - Vouzon were aligned using the Clustal Omega alignment program implemented in Seaview 4.5.0 (16). Nucleotide sequences were translated into amino-acid sequences and analysed using SeaView 4.5.0.

HTS method

For HTS analysis, the PUUV S segment (1750 bp) was sequenced using MiSeq Illumina technology with 10 overlapping amplicons (named A to J) of about 250 bp (see Supplementary Table S3). To do so, 300 ng of viral RNA from supernatant of Vero E6 cells and 1500 ng from lung tissue of infected rodents were reverse-transcribed using the SuperScript III First-Strand Synthesis System (Invitrogen) according to the manufacturer's instructions. The reverse-transcription reaction was performed with 2 µM of primers PUU1F1 (5'-CCTTGAAAAGCTACTACGAG-3') and PUU1R1 (5'-CCTTGAAAAGCAATCAAGAA-3') and with 50 ng of random hexamers provided in the kit. The sequencing libraries were prepared using a two-step PCR strategy adapted from Galan et al. (14) (see Supplementary information S4) and combined in a multiplex sequencing approach using unique dual indices (UDI) (18): each 9-bp i5 and i7 dual index was used only for one PCR sample without combinatorial indexing to ensure that libraries were sequenced and demultiplexed with the highest accuracy without any "leakage" between libraries (19). The libraries were sequenced on a MiSeq platform (GenSeq, Montpellier, France) with a 500-cycle reagent kit v2 (Illumina). A run of 250 bp paired-end sequencing was performed. The sequences were analysed with the data pre-processing tool FROGS (Genotoul) (20) and chimeric variants were removed using the isBimeraDenovo function of Dada2 R package (21). Each sample was analysed independently (qRT-PCRs, PCR amplifications and sequencing) using at least three PCR replicates to distinguish the true genetic variants and the artefactual mutations due to polymerase or sequencing errors. Mutations below a threshold of 0.32% were removed (see Supplementary information S5).

Supplementary Table S2: PUUV sequences obtained in this study

Strain or isolate† name	GenBank Accession No (S segment)	GenBank Accession No (M segment)	Sampling Year	GenBank Host
Hargnies-Mg	MW148463	MW148469	2018	*Myodes_glareolus*
Vouzon-Mg	MW148464	MW148470	2018	*Myodes_glareolus*
Hargnies-E6	MW148465	MW148471	2017	Vero_E6_cells
Ard161_Ardennes_Mg_2011†	MW148466	MW148472	2011	*Myodes_glareolus*
Vouzon-E6	MW148467	MW148473	2017	Vero_E6_cells
NCHA000357_Loiret_Mg_2014 †	MW148468	MW148474	2014	*Myodes_glareolus*

Supplementary Table S3: Sequences of primer cocktails used in Miseq

Amplicons	Primers names	Primer sequences (5' to 3')
A	PUMAMISEQ-Abis-F	GAGGATATAACCCGCCATGAA
	PUMAMISEQ-Abis1-F	GAGGTTATAACCCGCCATGAA
	PUMAMISEQ-Abis2-F	GAAGATATAACCCGCCATGAA
	PUMAMISEQ-A1-R	CAGTCGGGTCAGTAGGCTTA
	PUMAMISEQ-A2-R	CAGTCGGGTCAGCAGGCTTA
B	PUMAMISEQ-B1-F	TGGARGACAAACTTGCA
	PUMAMISEQ-B2-F	TGGARGATAAACTTGCA
	PUMAMISEQ-B1-R	ATRATAGGGAGTGTAAAGCC
	PUMAMISEQ-B2-R	ATRATAGGGAGTGTGAAGCC
	PUMAMISEQ-B3-R	ATRATAGGGAGTGTAAATCC
C	PUMAMISEQ-C1-F	GCCAAACAGCAGACTGGTA
	PUMAMISEQ-C2-F	GTCAAACAGCAGACTGGTA
	PUMAMISEQ-C3-F	GACAAACAGCAGATTGGTA
	PUMAMISEQ-C1-R	GTYARYTCYTCTGCTTTCATGGT
	PUMAMISEQ-C2-R	GTYARYTCYTCAGCCTTCATAGT
	PUMAMISEQ-C3-R	GTYARYTCYTCCGCCTTCATAGT
	PUMAMISEQ-C4-R	GTYARYTCYTCTGCCTTCATAGT
	PUMAMISEQ-C5-R	GTYARYTCYTCTGCTTTCATAGT
D	PUMAMISEQ-D1-F	GCATTAGAAGACCAAAGCACTT
	PUMAMISEQ-D2-F	GTATCAGGAGACCAAAGCATCT
	PUMAMISEQ-D3-F	GTATCCGAAGACCATAGCACTT
	PUMAMISEQ-D4-F	GTATTAGAAGACCAAAGCATTT
	PUMAMISEQ-D5-F	GTATTCGCAGGCCAAAGCATCT
	PUMAMISEQ-D6-F	GTATTCGCAGGCCAAAGCACCT
	PUMAMISEQ-D1-R	GGTGTBCCCGGTTTTAT

	PUMAMISEQ-D2-R	GGTGTBCCTGGCTTCAC
	PUMAMISEQ-D3-R	GGTGTBCCGGGTTTCAC
	PUMAMISEQ-D4-R	GGTGTBCCCGGTTTCAT
	PUMAMISEQ-D5-R	GGTGTBCCTGGCTTGAC
	PUMAMISEQ-D6-R	GGTGTBCCTGGTTTGAC
	PUMAMISEQ-D7-R	GGTGTBCCTGGATTGAC
E	PUMAMISEQ-E1-F	TBAARGAYTGGGCAGACCGGA
	PUMAMISEQ-E2-F	TBAARGAYTGGGCAGATCGGA
	PUMAMISEQ-E3-F	TBAARGAYTGGACAGATCGGA
	PUMAMISEQ-E4-F	TBAARGAYTGGACAGACCGGA
	PUMAMISEQ-E1-R	CARGGTGCATTAGGAGAC
	PUMAMISEQ-E2-R	CARGGTGCATTTGGGGCT
	PUMAMISEQ-E3-R	CARGGTGCATTGGGTGAC
	PUMAMISEQ-E4-R	CARGGTGCATTAGGGGAC
F	PUMAMISEQ-F1-F	TYGAYTAYGCAGCCTCAGGA
	PUMAMISEQ-F2-F	TYGAYTAYGCAGCTTCCGGA
	PUMAMISEQ-F3-F	TYGAYTAYGCAGCCGCAGGA
	PUMAMISEQ-F1-R	GRGTYCKTCGCAGGTATG
	PUMAMISEQ-F2-R	GRGTYCKCCGCAGGTATG
	PUMAMISEQ-F3-R	GRGTYCKTCGCAAGTATG
	PUMAMISEQ-F4-R	GRGTYCKTCTTAGGTATG
G	PUMAMISEQ-G1-F	CAGGAYATGAGGAATACCATC
	PUMAMISEQ-G2-F	CAGGAYATGAGAAATACCATC
	PUMAMISEQ-G3-F	CAGGAYATGAGAAACACCATC
	PUMAMISEQ-G4-F	CAGGAYATGAGGAATACTATC
	PUMAMISEQ-G5-F	CAGGAYATGAGGAACACCATC
	PUMAMISEQ-G1-R	TYARGGGTTCCTGATTAGAA
	PUMAMISEQ-G2-R	TYARGGGCTCCTGGTTGGAG
	PUMAMISEQ-G3-R	TYARGGGCTCTTGATTGGAT
	PUMAMISEQ-G4-R	TYARGGGTTCCTGATTGGAA
	PUMAMISEQ-G5-R	TYARGGGCTCCTGGTTTGAT
	PUMAMISEQ-G6-R	TYARGGGCTCCTGCTTGGAG
H	PUMAMISEQ-H1-F	ATGGTRGAYCACTTTCATCTG
	PUMAMISEQ-H2-F	ATGGTRGAYCATTTCCACCTT
	PUMAMISEQ-H3-F	ATGGTRGAYCACTTTCACCTG
	PUMAMISEQ-H4-F	ATGGTRGAYCACTTTCATTTG
	PUMAMISEQ-H1-R	AGTTAAACCCTGATTAATCTAA
	PUMAMISEQ-H2-R	AGTTAAACCCTGATTAACCTGA
	PUMAMISEQ-H3-R	AGTTAAACCCTGATCAACCTAA
	PUMAMISEQ-H4-R	AGTTAAACCCTGATTGACCTTA
I	PUMAMISEQ-I1-F	GGGATTAYYDTAATTAATTGTT
	PUMAMISEQ-I2-F	GGGATTAYYDTAGTTAATTGTT
	PUMAMISEQ-I3-F	GGGATTAYYDTAGTTAAGTGTT

	PUMAMISEQ-I4-F	GGGATTAYYDTAGTTGAATGTT
	PUMAMISEQ-I1-R	GCTCAGTTTCACATTATTGG
	PUMAMISEQ-I2-R	GCTTAGTTTCACATTATTGG
	PUMAMISEQ-I3-R	GCTCAGTTTCACATTAATGG
J	PUMAMISEQ-J1-F	TGCTGCTTAATCATTATTACCAGCA
	PUMAMISEQ-J2-F	TGCTGCTCAATTATTATCACCAGCT
	PUMAMISEQ-J3-F	TGCTATTGATAAGTGTTACCAGCA
	PUMAMISEQ-J4-F	TGCTGCTTAATCATTATACCCAGCA
	PUMAMISEQ-J5-F	TGCTGCTTGATTATTGTAACCAGCA
	PUMAMISEQ-J1-R	GAAATCAGTATGTTGAGGTAGT
	PUMAMISEQ-J2-R	GAAATCGGCATGTTGAGGTAGT

Supplementary information S4: Library preparation adapted from Galan et al. 2018

PCR1 was performed in a 10 µL reaction volume using 5 µL of 2X Qiagen Multiplex Kit Master Mix (Qiagen), 0.3 µM of each primer and 2 µL of 4-fold diluted cDNA. PCR conditions consisted of an initial denaturation step at 95 ℃ for 15 min, followed by 40 cycles of denaturation at 94 ℃ for 30 s, annealing at 50 ℃ for 1 min, and extension at 72 ℃ for 40 s, followed by a final extension step at 72 ℃ for 10 min.

The PCR2 consisted of a limited-cycle amplification step to add multiplexing indices i5 and i7 and Illumina sequencing adapters P5 and P7 at both ends of each cDNA fragment. PCR2 was carried out using 5 µL of 2X Qiagen Multiplex Kit Master Mix, 0.5 µM of each indexed primer and 2 µL of PCR1. Amplification started with an initial denaturation step of 95 ℃ for 15 min, followed by 8 cycles of denaturation at 94 ℃ for 40 s, annealing at 55 ℃ for 1 min and extension at 72 ℃ for 1 min, followed by a final extension step at 72 ℃ for 10 min.

The average expected size of the super pool for excision on an agarose gel was 406 bp (including primers, indices and adaptors). xx 12 pM of library and 5% of PhiX control were loaded on a Miseq flow cell.

Supplementary information S5: Definition of the 0.32% threshold

Because plasmid DNA propagation has a much lower error rate (≈10^{-7}–10^{-8} errors/site/copy) than RNA virus genome replication, a plasmid-generated "viral" RNA can be considered as an extremely low diversified starting population. This strategy can be used to determine the error rate and separate real mutations from process error (29).

In this study, the threshold of 0.32% was set after sequencing a plasmid generated from the Sotkamo PUUV strain. First, the complete PUUV S segment region (1759 bp) was amplified in 10 replicates with a final volume of 10 µL. We used 5 µL of 2X Qiagen Multiplex Kit Master Mix, 0.3 µM of forward PUMAEXTLG-F (5'-CTGGAATGAGTGACTTGACAG-3') and reverse PUMAEXTLG-R (5'-CAGCATGTTGAGGTAGTATATTG-3') primers and 2 µL of 5-fold diluted Sotkamo cDNA. PCR conditions consisted in an initial denaturation step at 95 °C for 15 min, followed by 40 cycles of denaturation at 94 °C for 30 s, annealing at 45 °C for 90 s, and extension at 72 °C for 2 min, followed by a final extension step at 72 °C for 10 min. Then, 5 µL of PCR product was verified by electrophoresis on a 1.5% agarose gel. Then, all PCR products were pooled, purified with Ampure XP beads (Beckman coulter) and quantified with Qubit Fluorometer (ThermoFischer).

PCR products were cloned using the pGEM-T Easy vector system 2 (Promega) following the manufacturer's instructions. A 3:1 PCR product: vector molar ratio was used for ligation. Transformation was performed using JM109 High Efficiency competent cells growing on LB/Ampicillin/IPTG/X-Gal Petri plates overnight at 37 °C. White bacterial colonies were picked and screened by PCR amplification using T7 and SP6 primers. Two recombinant plasmids were selected and purified using Qiaprep Spin Mini Prep (Qiagen) and then sequenced with Sanger technology at Eurofins MWG. Finally, 50-fold dilutions of the two purified plasmid were used as template for short amplification (Illumina) to define the cycle threshold.

Supplementary information S6: Means number of read used polymorphic sites quantification

PUUV origin / bank vole number	Strains	Tissue	Mean Nb of reads *(amplicon after data filtering)*
in natura	Hargnies	Lungs	95 477
in natura	Vouzon	Lungs	131 769
in vitro	Hargnies	Cell culture	186 547
in vitro	Vouzon	Cell culture	112 711
in vivo / 811	Hargnies	Lungs	32 360
in vivo / 812	Hargnies	Lungs	33 484
in vivo / 791	Vouzon	Lungs	31 935
in vivo / 792	Vouzon	Lungs	32 248
